# MOrtality and infectious complications of therapeutic E*ndo*VA*scular interventional radiology:* a systematic and meta-analysis protocol

**DOI:** 10.1186/s13643-017-0474-y

**Published:** 2017-04-24

**Authors:** Kaoutar Mellouk Aid, Hervé Tchala Vignon Zomahoun, Abdelmajid Soulaymani, Karin Lebascle, Stephane Silvera, Pascal Astagneau, Benoit Misset

**Affiliations:** 1 Laboratory of Genetics and Biometrics, Faculty of Sciences, Ibn Tofail University, Kenitra, Morocco; 2 Clinical Research Centre, Foundation Hospital Saint-Joseph, 185 Rue Raymond Losserand, 75014 Paris, France; 3 Québec SPOR-SUPPORT Unit Research Centre of CHU de Québec, Université Laval, Québec City, Québec Canada; 4 Centre for Control of Healthcare-Associated Infections, Paris, France; 5 Foundation Hospital Saint-Joseph, 185 Rue Raymond Losserand, 75014 Paris, France; 6 Centre for Control of Healthcare-Associated Infections and Pierre & Marie Curie Faculty of Medicine, Sorbonne Universities, Paris, France; 7 Department of Intensive Care and Clinical Research Centre, Foundation Hospital Saint-Joseph, 185 Rue Raymond Losserand, 75014 Paris, France; 8 Paris Descartes University, Paris, France; 9 Department of Intensive Care, Rouen, France; 10 Rouen University Hospital, University of Rouen, Rouen, France

**Keywords:** Endovascular interventional radiology, Risk of complications, Infectious complications, Epidemiology, Mortality, Systematic review, Meta-analysis

## Abstract

**Background:**

Endovascular interventional radiology (EIR) is an increasingly popular, mini invasive treatment option for patient with symptomatic vascular disease. The EIR practiced by qualified hands is an effective, well-tolerated procedure that offers relief of patient’s symptoms with a low risk of complications. During acute post procedural period, immediate complications may relate to vascular access, restenosis, thromboembolic events, uterine ischemia, infection, necrosis, sepsis, ICU stay, surgical recovery, pain management, treatment failure, and death. Moreover, additional non-life-threatening complications exist, but they are not well described and represent disparate information.

**Methods/design:**

A range of databases will be screened consulted to identify the relevant studies: PubMed, EMBASE, The Cochrane Library, NosoBase, and Google Scholar (to identify articles not yet indexed). Scientist librarian used Medical Subject Headings (MeSH) and free terms to construct the search strategy in PubMed. This search strategy will be adapted in other databases. Two coauthors will independently select the relevant studies, extract the relevant data, and assess the risk of bias in the included studies. Any disagreements between the two authors will be solved by a third author.

**Discussion:**

This systematic review will provide a synthesis of EIR complications. The spotlighted results will be analyzed in order to provide a state-of-knowledge synopsis of the current evidence base in relation to the epidemiology of the infectious complications after EIR. In the event of conclusive results, our findings will serve as a reference background to assess guidelines on reality of the problem of the infections linked to endovascular interventional radiology and to formulate of assumptions and propose preventive measures, based on the results of our investigations. These propositions will aim to reduce the risk and/or the severity of these complications in the concerned population in favor a positive medical economics report. It will also aim to decrease the antibio-resistance and in fine will improve health status and security of patients.

**Systematic review registration:**

PROSPERO CRD42015025594

**Electronic supplementary material:**

The online version of this article (doi:10.1186/s13643-017-0474-y) contains supplementary material, which is available to authorized users.

## Introduction

Interventional radiology is a clinical discipline with a procedural foundation rooted in diagnostic imaging and almost entirely innovation dependent. Interventional radiologists possess a special blend of knowledge based on a fundamental diagnostic imaging complemented by technical and clinical management expertise when applied skillfully and with care, can save and improve lives cost-effectively [1].

Since the first interventional radiology held on January 16, 1964, on dilation of the stenosis with a guide wire and coaxial Teflon catheters, a catheter-based therapies, featuring low-risk expectation, low cost, and excellent outcomes [2, 3]. Currently, the interventional radiology involves biopsy of deep internal organs, drain abscesses and cysts, and open blocked arteries and veins with devices or medications. It does provide a way obstructed kidneys and biliary tracts might be emptied. Rather than an area of screening, interventional radiology is usually used for patients end up for therapeutic procedures, often after several other diagnostic studies [4]. The advantages of interventional radiology include [5, 6] (i) the ability to perform most procedures in an outpatient setting, (ii) general anesthesia is usually not required, (iii) risk, pain, and recovery time are often significantly reduced, and the procedures are sometimes less expensive than surgery.

Among these procedures, evaluation of cardiac status and coronary catheter arterial and selective angiography took a growing place in the assessment of prognosis of cardiovascular diseases. The angioplasty coronary revascularization method became the most used in the world [6]. Although the long-term durability of many of these interventional procedures remains to be established, it is estimated that in the near future, 40 to 70% of vascular interventions will be endovascular procedure [7]. As IR has been shown to be generally more cost-effective than alternative surgical treatments, there has been a tremendous increase in the number of IR procedures performed worldwide [8]. Due to the rapidly broadening spectrum of interventional percutaneous procedures, the trans-femoral access route is expected to remain a cornerstone of catheter-based diagnosis and treatment and valid alternative to the trans-radial access [9, 10]. However, complications of the vascular access site are still challenging representing the leading cause of morbidity associated with trans-femoral catheterization [11, 12].

Medical errors may occur in any medical field despite the safety of the procedure. In endovascular interventional radiology, a number of studies assessing the risk of adverse events to 0.06% for percutaneous catheterization, 0.64% for coronary angioplasty, and 4.9% for all arterial and venous angioplasty [11, 13]. A 2.6% of hepatic abscess rates have been reported after chemoembolization [14]. Bacteremia was observed in 35% of patients; including 13% of clinical infection, after completion of a trans-jugular intrahepatic porto-systemic shunts (TIPS) [15].

In endovascular interventional radiology, which exempts of vascular catheterization techniques, the number of infections varies also according to the procedure. Regarding guided percutaneous liver puncture, available figures vary from 0 to 0.3%, depending on the therapeutic actions of tumor destruction (radio-frequency) [16, 17].

In the endo-cavitary interventional radiology echo-guided, four observations of *Pseudomonas aeruginosa* infections have been reported after trans-rectal biopsy echo-geared [18]; the rate of infectious complications is between 3 and 10% [19].

For percutaneous gastrostomies, the risk of local infection would be higher with a radiological approach than an endoscopic approach (7.3 vs 1.%); however, antibiotic prophylaxis practices have different outcomes [20].

Some patients show up with a history of previous reaction to contrast agents. Contrast products are associated with a very low incidence of adverse reactions. According to Hunt et al., from 2002 to 2006, a global retrospective review of adverse effects of administration of low-osmolar iodinated and gadolinium contrast agents, a total of 456,930 contrast doses were administered. A merely 522 adverse effects were identified (0.114% of all doses). One death occurred 30 min after agent injection [21].

Complications related to interventional radiology are a part of the new subsisting public health problems. To our knowledge, no meta-analysis was conducted on this subject. Referring to the national French surveillance system for control of nosocomial infections, very few notifications of infectious complications have been reported for the last 10 years. Hence, we decided to perform a systematic review and meta-analysis to determine the state of the art on morbidity related to IR complications.

## Objective

This study aims to assess the associations between the endovascular interventional radiology and the infectious complications in children and adults.

## Methods/design

In the present protocol, we designed a systematic review and meta-analysis using the Cochrane Handbook recommendations [22]. This protocol was registered (CRD42015025594) in the Prospective Register of Systematic Reviews (PROSPERO) conforming the guidelines of Preferred Reporting Items for Systematic review and Meta-Analysis Protocols (see PRISMA-P checklist, Additional file 1) [23].

## Eligibility criteria

### Participant/population

This review will consider all studies conducted among children or adults. There will be no limitation on gender or health status (Additional file 2).

### Type of exposure

The exposure that will be assessed is aortic endovascular interventional radiology procedures for therapeutic purpose: angioplasty, therapeutic angiography, endoprosthesis, insertion of shunts and stents, embolization and thrombectomy, and aortic aneurysm therapy.

## Comparators

People who were not exposed to aortic endovascular interventional radiology will be considered for comparator groups.

### Type of outcomes

An infection is nosocomial if it appears during or following a hospitalization and if it was absent at admission at hospital [24]. For the infections of operational site and interventional radiology, one regards infection bound that occurred in the 30 days following the intervention, or, if there were installation of prosthesis, stent, or an implant, for the year which follows the intervention.

Secondary outcomes: the rate of technical failure, mechanical complications, length of hospitalization in intensive care unit, inefficiency of endovascular therapeutic interventions, surgical replacement therapy, and, finally, death frequency.

Only studies which feedback relative information about all post radiological infectious interventions will be included in this review. All infections (bacterial and fungus infections) will be considered. Studies in which procedures for diagnosis only reported will be excluded.

## Timing

We will consider only studies in which the nosocomial infection was diagnosed after a radiological act.

### Types of studies

The review will consider all experimental (randomized controlled trials) and observational studies (prospective and retrospective cohort studies).

## Language

We will consider articles published in French and English language.

## Period

There will be no restriction regarding publication date of studies. We will consider for screening all published studies until literature search date in different databases.

## Exclusion criteria

Articles that meet the following criteria will not be included in the review:

1. Literature reviews. However, the list of bibliographic references of relevant reviews will be screened to identify additional relevant studies.
2. Non-human study
3. Case reports or studies with less than three cases for each group
4. Scientific correspondence
5. Exposure related to chemoembolization
6. Exposure related to radiological techniques of diagnosis
7. Exposure related to surgical treatment only
8. Exposure related to radiological technique in a non-vascular territory (e.g, biliary tract, gastrointestinal tract, and bronchi)
9. Exposure related to secondary infection indirectly related to the arterial act (e.g, lung infection by inhalation and infection related to a digestive ischemia
10. Exposure related to absence of complications
11. Exposure related to central catheters with implantable chambers or not
12. Any infections preceding radiological act

### Search strategy

The search strategy will be designed to access both published and unpublished studies. To identify published studies, we will perform a systematic literature search using electronic databases: PubMed, EMBASE, The Cochrane Library, NosoBase, and Google Scholar. Our scientist librarian built a preliminary literature search strategy in PubMed using keywords related to exposition and main outcome (Additional file 2). The search terms will be adapted for the different databases using a combination of Medical Subject Heading (MeSH), free terms, and relevant keywords.

The equation of search was based on the following logic: Elements appropriate to the aortic endovascular interventional radiology «AND» Elements appropriate to infectious complications.

Additional studies will be sought in the bibliographic reference lists of studies selected from electronic databases and those of relevant systematic reviews. To identify unpublished studies, we will search through gray literature including reports and conference abstract books, according to diagramme of "Search strategy, description of data" (Additional file 3). We will also search ongoing trials in ClinicalTrials.gov and controlled-trials.com. Furthermore, the following data sources will be consulted:

- Society of Interventional Radiology
- Society of Pediatric Interventional Radiology
- Western Angiographic & Interventional Society
- The Interventional Initiative
- The Endovascular Forum
- Interventional radiology procedures

## Study selection process

Two independent reviewers (K. Mellouk and B. Misset) will initially screen primary titles and abstracts to select potential full text articles for further scrutiny. When the title and abstract is not rejected by any reviewer, the full text of the article will be obtained and will carefully assess for inclusion by the two reviewers (KM and BM).

For this step, we will adopt the selection form including the selection criteria (Additional file 4). We will pilot this form to ensure that the criteria are clear for the both reviewers. The pilot test of the selection criteria will be made on 10% of total unique references. The selection form would be amended/updated depending on the pilot test results.

## Data collection process

We will create an extraction data form and a codebook in which the relevant variables will be described (definition and modalities). A pilot of the data extraction form will be undertaken by two reviewers using a random sample representing 10% of included articles. The data extraction form and the codebook will be amended/updated as necessary.

One author (KM) will extract data from all the included papers and another coauthor (BM) will verify the accuracy of this extraction. Any disagreement will be solved through discussion. If no agreement is reached, a third author (PA) will be consulted. Disagreements between KM, BM, and PA will be resolved by consensus, according to diagramme of "Step of selection of the articles/screening" (Additional file 5).

The following pre-specified variables will be extracted from selected studies:

a Characteristic of studies and participants
  First author’s name
  Country, city, and hospital where patients recruited
  Year of publication
  Year of patient recruitment (the midpoint of the study’s time period)
  Study design
  Type of participants’ sample (e.g., consecutive and random)
  Sample size
  Rate of participation
  Study setting
  Socio-demographic characteristics of participants (age, sex, race/ethnicity, education level, socio-economic level, and other existing diseases)
  EIR history
b Characteristics of intervention
  EIR urgently performed or not
  EIR technique used
  Description of EIR procedure-type of endovascular site of intervention
  Type of catheter used
  Method used to sterilize the intervention equipment
  Reasons of EIR
  Duration of EIR
  Observation time post EIR
  Number of monitoring visits after EIR
  Intervention team profile (e.g., healthcare assistant, nurse, technologist, physician, and/or radiologist)
  Interventionist’s profile (e.g., sex, age, time since graduation, and experience in EIR)
c Characteristics of outcomes
  Nosocomial infection (infectious agent name, site, type, and diagnostic test used)
  Other complications
  Technical failure
  Mechanical complications
  Hospitalization in intensive care unit (ICU)
  Inefficiency of endovascular therapeutic interventions
  Hospitalization for second time
  Surgical recovery
  Death
d Effect of endovascular interventional radiology on outcomes
  Dichotomous outcomes
    Definition and measurement of outcome
    Number of events and sample size in each group
    Non-events and sample size in each group
    Events and non-events in each group
    Event rate and sample size in each group
    Odds ratio (OR), risk ratio (RR), or hazard ratio (HR), 95% confidence interval (CI), and *p* value
  Continuous outcomes
    Definition and measurement of outcome
    Mean, standard deviation (SD), and sample size in each group
    Difference of means, 95% CI, and *p* value

## Assessment of risk of bias in individual studies

The goal of this assessment is to make a methodological judgment of whether the design and implementation of the study compromised the internal validity of the association between exposure (therapeutic endovascular interventional radiology) and the outcome (infectious complications or other clinical outcomes). The presence of potential bias within individual studies will be assessed independently by the two authors (KM and BM) using the Cochrane’s “risk of bias” assessment tool for randomized controlled trials [23] and the Newcastle-Ottawa Scale for observational studies [25].

Only studies of good and average quality will be retained for statistical analysis.

## Data synthesis and analysis

Only studies, in which data on endovascular interventional radiology and outcomes will be available to estimate the effect size, will be included in the meta-analysis. When effect sizes will not be calculable or when only one effect size available for an outcome, we will report the results of this outcome as a narrative synthesis. If effect sizes will be available or calculable in two or more studies for a specific outcome, meta-analysis will be conducted using the software Review Manager (RevMan). We will use, as effect sizes, the RR with 95% CI for dichotomous outcomes and standardized mean difference (SMD) with 95% CI for continuous outcomes. Since we anticipate a potential heterogeneity in studies, we will use a random-effects model to pool effect sizes of endovascular interventional radiology for each outcome [26]. Only the adjusted effect size will be considered in this model. We will also calculate Higgins’ *I* ^2^ statistic that is the percentage of variability in the effect size estimates due to the heterogeneity [27]. The chi-squared test will be used to test the heterogeneity [28].

Moreover, the potential heterogeneity will be explored using subgroup analyses based on studies, participants, and intervention characteristics mentioned above.

We will also assess the publication bias for each outcome by visually examining funnel plots when more than ten studies will be included in the meta-analysis.

To assess the robustness of our results, we plan to perform a few sensitivity analyses. First, we will explore the individual influence of each study by removing one at the time from the pooled effect size estimation. Second, we will repeat the pooled effect size estimation for each outcome by including only studies with low risk of bias. Finally, we anticipate that confounding variables could vary according to studies.

Thus, we will estimate the pooled crude effect size for each outcome using crude effect sizes (non-adjusted) only. Results of these analyses will be compared with initial pooled effect size in order to assess the confounding variables’ impact.

## Confidence in cumulative evidence

In order to reduce the misinterpretation of our review’s results, we will assess, for each outcome, the quality of cumulative evidence with the Grading of Recommendations Assessment, Development and Evaluation (GRADE) [29]. This tool is based on five criteria such as individual study risk of bias, indirectness of the evidence, data heterogeneity, imprecision of the effect size estimates, and risk of publication bias. For each outcome, the quality of evidence will be rated high, moderate, low, or very low.

## Discussion

Therapeutic endovascular interventional radiology through a percutaneous route is increasing rapidly in volume, and their potential adverse effect may rise accordingly.

This protocol will allow to perform a systematic review of the epidemiology of these events. This will serve as the rational for designing future epidemiological and interventional studies.

Interventional or therapeutic radiology is now a discipline that achieves a complete support from patients. This efficient multidisciplinarily gains confirmation and strength from health technical developments leading to new frontiers between exploration (imaging, biology, and functional) and treatment techniques (interventional imaging, endoscopy, surgery, therapy by physical agents, and the evolutions of the patients demand, for a supported rapid, effective, and less invasive technique [30].

The patient’s condition sent in IR is extremely variable depending on their department of original (intensive care, for example), their age, subjacent pathologies, the evolving affection condition, the existence of factors promoting the infection, their immune status, the presence of invasive devices (catheters and probes), skin lesions, the presence or not of infection, or known portage or not of microorganisms such as multiresistant ones to antibiotics (BMR bacteria epidemic-prone (such as MRSA and ESBL enterobacteria) and bacteria highly resistant to antibiotics (BHR, such as GRE and CPE). In addition, IR procedure usually performed in a hospital setting increases the risk of exposure to blood and biological fluids for the professional staff [31].

The acts charged are extremely varied, with very different risks. Group SFR - IRF (French society of Radiology - Interventional Radiology Federation) has established a list of acts of IR, classifying them into three categories, depending on the level of complexity, including potential risks, particularly, the risk infectious [32]. For each category, a number of precautions are required, the level of infectious risk determining the level of precautions to be taken [32].

Control of the risk of infection in IR is thus based on the strict observance of standard hygiene precautions and the existence of procedures defined, applied and evaluated, maintenance of the various equipment, in particular, those used for guidance, in an integrated environment [33, 34].

Although there are no controlled trials to establish formal evidence of an advantage of prophylactic antibiotics in IR, it should be considered in certain situations [35–37]. Its use must be weighed against the potential risks of misuse (germ resistant and hypersensitivity selection) and was decided in a multidisciplinary way [38]. It should be the subject of a service protocol, referring to the consensus conference of the French Anesthesia and Reanimation Society (SFAR), who, under the aegis of the French National Authority for Health (HAS) and in collaboration with the concerned societies and in particular the IRF, proceeded, in 2010, the update of the perioperative recommendations in surgery and interventional radiology [39]. Prophylaxis antibiotic therapy is recommended for endoscopic gastrostomies, sclerosis of varicose veins of the esophagus, stents, and stents (except intra-coronal); it should be also considered in some subjects at risk [40].

The risk of other complications over the procedure of the IR according to some studies is linked to a volume effect (the relationship between the volume of activity and the therapeutic risk incurred). Many studies have evaluated the relationship between coronary angioplasty activity level and the risk of serious complications (death of the patient and need for bypass in emergency of myocardial infarction) due to therapeutic procedures [41]. These publications show that the risk for a patient is inversely proportional to the level of activity of the center in which it is processed [6].

The physician experience performing angioplasty has a similar impact; especially when a large majority of patients with complex lesions are treated with this technique of revascularization [42–45]. After adjusting for other risk factors, this study highlights a strong correlation between the number of patients treated annually by angioplasticien and the reduction of risk of serious cumulative complications (myocardial infarction, aorta-coronary bypass in emergencies, and death).

The angioplasticiens dealing with less than 70 patients per year have an overall rate of serious complications of 9.3%. While the angioplasticiens operating more than 270 patients per year, the rates are respectively 2.9 and 1.7% (*p* < 0.001). The risk of angioplasty decreased up to a threshold between 225 and 270 annual angioplasties by doctor [46]. Beyond that, the rate of complications reached a remarkably low level, despite the more serious patient’s support [47].

## Additional files

Additional file 1: PRISMA-P file, PRISMA-P 2015 Checklist. (DOCX 38 kb)

Additional file 2: Population, intervention, comparator, outcomes, study design, and time breakdown of study eligibility criteria. (DOC 31 kb)

Additional file 3: Search strategy, description of data. (DOC 25 kb)

Additional file 4: Decisional diagram of selection of an article starting from the title and summary. (DOC 53 kb)

Additional file 5: Step of selection of the articles/screening. (DOC 27 kb)

## Abbreviations

BHR: Bacteria highly resistant
BMR: Bacteria Multi-Resistance
CI: Confidence interval
CPE: Carbapenemase-producing enterobacteria
EIR: Endovascular interventional radiology
ESBL: Extended-spectrum beta-lactamases secreting enterobacteria
GRE: Glycopeptide-resistant enterococci
HAS: French National Authority for Health
HR: Hazard ratio
ICU: Intensive care unit
IRF: Interventional Radiology Federation
MRSA: Methicillin-resistant *Staphylococcus aureus*
OR: Odds ratio
RR: Risk ratio
SD: Standard deviation
SFAR: French Anesthesia and Reanimation Society
SFR: French Society of Radiology
SMD: Standardized mean difference
TIPS: Trans-jugular intrahepatic porto-systemic shunts IR
