# Supplementary material for: MOrtality and infectious complications of therapeutic EndoVAscular interventional radiology: a systematic and meta-analysis protocol
Source: Syst Rev. 2017 Apr 24;6:89. doi: 10.1186/s13643-017-0474-y (PMC5402637; doi:10.1186/s13643-017-0474-y)
Supplement: Supplementary file 2 — Population, intervention, comparator, outcomes, study design, and time breakdown of study eligibility criteria. (DOC 31 kb) [file 13643_2017_474_MOESM2_ESM.doc]

***Additional file*** 2***;*** Population, intervention, comparator, outcomes, study design, and time breakdown of study eligibility criteria

| **Category** | **Description of criteria** |
| --- | --- |
| Population | All patients are children’s and adults. There were no limitations on gender, or health status. |
| Exposure | Arterial therapeutic endo-vascular interventional radiology procedures: angioplasty, endo-prosthesis, Insertion of shunts and stents, embolization, thrombectomy and aortic aneurysm therapy. |
| Comparator | People who were not exposed to aortic endo vascular interventional radiology will be considered for comparator groups. |
| Outcomes | Primary outcome   - Infectious complications after endovascular interventional radiology. All infections (bacterial, fungus and viral infections) will be taken into account.   Secondary outcomes  - Other complications  - Technical failure  - Mechanical complications  - Hospitalization in Intensive care unit ICU  - Inefficiency of endovascular therapeutic interventions,  - Hospitalization for second time,  - Surgical recovery  - Death, |
| Study design | All experimental (randomized controlled trials) and observational studies (prospective and retrospective cohort studies mainly with consecutive patient sampling). |
| Time | We will consider only studies in which the nosocomial infection was diagnosed after a radiological act. For the infections of operational site and interventional radiology one regards infection bound that occurred in the 30 days following the intervention, or, if there were installation of prosthesis, stent or an implant, for the year which follows the intervention. |
