# Supplementary material for: MOrtality and infectious complications of therapeutic EndoVAscular interventional radiology: a systematic and meta-analysis protocol
Source: Syst Rev. 2017 Apr 24;6:89. doi: 10.1186/s13643-017-0474-y (PMC5402637; doi:10.1186/s13643-017-0474-y)
Supplement: Supplementary file 3 — Search strategy, description of data. (DOC 25 kb) [file 13643_2017_474_MOESM3_ESM.doc]

***Additional*** file 3: Search strategy, Description of data.

The classification of therapeutic endovascular interventional radiology acts was established according to the society of French radiology (Federation of Interventional Radiology 2012).

Each act was translated into the French Catalogue of the Medical sites indexation (CISMEF). For the consultation of the MESH and other terminologies, we consulted the Health terminology – Ontology Portal (HETOP).

The research equation was established according to the thesauri of each database, using keywords and/or free text.

**The logic of the search equation**:

[Interventional arterial radiology acts] AND [Infectious complications].

### Patient- Exposure criteria

### MESH

### (Radiology, Interventional OR Angiography/therapy OR Angioplasty OR Stents OR Embolization, Therapeutic OR Balloon Embolectomy OR Endovascular Procedures/therapy OR Thrombectomy OR Aortic Aneurysm, Thoracic/therapy)

### Coding free words [Title/Abstract]

(Interventional Radiology OR (Angiography AND Therapy) OR Angioplasty OR Stent* OR Therapeutic Embolization OR Balloon Embolectomy OR (Endovascular Procedure* AND arterial) OR Thrombectomy OR (Aortic Aneurysm AND Therapy)

### Outcome criteria

**MESH**

(Cross infection OR Disease transmission, infectious OR Surgical wound infection OR Bacteremia OR Sepsis OR Abscess OR [Disease Outbreaks](http://www.ncbi.nlm.nih.gov/pubmed))

### Coding free words [Title/Abstract]

(Cross infection* OR (Infectious disease AND transmission) OR Surgical wound infection OR Bacteremia OR Sepsis OR Abscess OR Surgical site infection OR Nosocomial infection* OR Hospital acquired infection OR (Puncture AND infection))

### Characteristic according to databases

PubMed search: We used the same terms in MeSH and coding free words. Search in free words was carried out by using the word plus star* at the end (without taking into account neither plural, nor singular). About to research of the infectious complications, we also added a few synonyms in free words.
