# Supplementary material for: MOrtality and infectious complications of therapeutic EndoVAscular interventional radiology: a systematic and meta-analysis protocol
Source: Syst Rev. 2017 Apr 24;6:89. doi: 10.1186/s13643-017-0474-y (PMC5402637; doi:10.1186/s13643-017-0474-y)
Supplement: Supplementary file 4 — Decisional diagram of selection of an article starting from the title and summary. (DOC 53 kb) [file 13643_2017_474_MOESM4_ESM.doc]

***Additional file 4:*** Decisional diagram of selection of an article starting from the title and summary

**Read title**

**Is title evocating interest of the article, compared to our aim of study?**

**Definitive rejection of the article**

**No to read article**

**Read summary**

**Checking of inclusion and exclusion criteria.**

**Retain article (the article is passed selection step)**

**Yes**

**And /Or**

**Answer by yes to all inclusion criteria**

**Incertain**

**No at only one criterion of exclusion**

**No at only one criterion of exclusion**
