# Supplementary material for: MOrtality and infectious complications of therapeutic EndoVAscular interventional radiology: a systematic and meta-analysis protocol
Source: Syst Rev. 2017 Apr 24;6:89. doi: 10.1186/s13643-017-0474-y (PMC5402637; doi:10.1186/s13643-017-0474-y)
Supplement: Supplementary file 5 — Step of selection of the articles/screening. (DOC 27 kb) [file 13643_2017_474_MOESM5_ESM.doc]

***Additional file 5:*** Step of selection of the articles / screening.

The articles which can be eliminated on the level of the reading of title

- Is that study related to animals?

 Yes, reject reference No, retain the reference and check other criteria

- Is that study related to chemo-embolization?

 Yes, reject reference No, retain the reference and check other criteria

- Is that article written in English, or in French?

Yes, retain the reference and check other criteria No, reject reference.

After provision of summary, some criteria can be defined

- Is that study related to a case report?

Yes, reject reference, No, retain the reference and check other criteria

- Is that study related to surgical Treatment only?

Yes, reject reference, No, retain the reference and check other criteria

- Is that study related to radiological techniques of diagnosis?

Yes, reject reference, No, retain the reference and check other criteria

- Does that study relate to central catheters with implantable chambers or not?

Yes, reject reference, No, retain the reference and check other criteria

- Is that study related to radiological technique in a non-vascular territory (ex: biliary tract, gastro-intestinal tract, bronchi…)

 Yes, reject reference, No, retain the reference and check other criteria

- Is that study related to secondary infection indirectly related to the arterial act (ex: lung infection by inhalation, infection related to a digestive ischemia, etc…)

Yes, reject reference, No, retain the reference and check other criteria

- Is that study related to absence of search for complications?

Yes, reject reference, No, retain the reference and check other criteria

Is Infection precedes radiological act? (The intervention is made to diagnosis or to treat the infection?)

Yes, reject reference, No, retain the reference and check other criteria
